# 低T3综合征对初诊多发性骨髓瘤患者预后的影响

**DOI:** 10.3760/cma.j.issn.0253-2727.2023.03.007

**Published:** 2023-03

**Authors:** 珏 张, 丽娜 张, 园 夏, 媛媛 金, 旭星 沈, 丽娟 陈

**Affiliations:** 南京医科大学第一附属医院，江苏省人民医院血液科，南京 210029 Department of Hematology, the First Affiliated Hospital of Nanjing Medical University, Jiangsu Province Hospital, Nanjing 210029, China

**Keywords:** 低T3综合征, 多发性骨髓瘤, 预后, Low T3 syndrome, Multiple myeloma, Prognosis

## Abstract

**目的:**

探讨低T3综合征（LT3S）与初诊多发性骨髓瘤（MM）患者预后的相关性。

**方法:**

回顾性分析2009年7月至2020年12月江苏省人民医院血液科诊治的211例初诊MM患者，所有患者均进行了甲状腺功能检查以评估是否合并LT3S。分析LT3S与临床特征的相关性及对预后的影响。

**结果:**

211例患者中男119例、女92例，中位年龄60（33～86）岁。与不伴LT3S患者相比，伴LT3S组患者初诊时β_2_-微球蛋白、C-反应蛋白和血肌酐水平明显升高（*P*值均<0.001），血红蛋白、血小板和血清白蛋白水平显著降低（*P*值分别为<0.001、0.018、<0.001），ISS分期Ⅲ期比例更高（*P*<0.001）。伴LT3S组较不伴LT3S组具有更短的中位无进展生存（PFS）期（16个月对 30个月，*P*＝0.003）和总生存（OS）期（57个月对75个月，*P*＝0.004）。在多因素分析中，LT3S是影响患者PFS（*HR*＝2.114，95％ *CI* 1.271～3.516，*P*＝0.004）和OS（*HR*＝2.231，95％ *CI* 1.088～4.577，*P*＝0.029）的独立不良预后因素。

**结论:**

LT3S和初诊MM患者预后不良显著相关。

## Abstract (en)

**Objective:**

This study aimed to examine the relationship between low T3 syndrome (LT3S) and the prognosis of newly diagnosed multiple myeloma (NDMM) patients.

**Methods:**

A retrospective examination of 211 NDMM patients treated at the Department of Hematology, Jiangsu Provincial People's Hospital from July 2009 to December 2020 was performed, and all patients received thyroid function testing to determine if they had LT3S. We investigated the relationship between LT3S and clinical features, as well as its impact on MM prognosis.

**Results:**

Of the 211 patients, 119 were males, and 92 were females, with a median age of 60 (33–86) years. Patients with LT3S had significantly higher levels of β_2_-microglobulin, C-reactive protein, and blood creatinine compared to those with normal T3 levels. They also had lower levels of hemoglobin, platelets, and serum albumin, as well as more advanced ISS stages (*P*<0.001). Patients with LT3S had shorter progression-free survival (PFS) (16 months *vs* 30 months, *P*＝0.003) and overall survival (OS) (57 months *vs* 75 months, *P*＝0.004) than patients without LT3S. LT3S was found to be a standalone unfavorable factor in multivariate analysis, LT3S was an independent unfavorable factor in predicting both PFS (*HR*＝2.114, 95％ *CI* 1.271–3.516, *P*＝0.004) and OS (*HR*＝2.231, 95％ *CI* 1.088–4.577, *P*＝0.029).

**Conclusion:**

Low T3 syndrome was an independent unfavorable prognostic predictor for NDMM.

多发性骨髓瘤（MM）是一种克隆性浆细胞异常增殖性疾病，其预后具有显著异质性[1]–[4]，因此，精确的预后分层对于指导治疗具有重要意义。低T3综合征（low T3 syndrome, LT3S）也被称为正常甲状腺病态综合征（euthyroid sick syndrome, ESS）或非甲状腺疾病综合征（nonthyroidal illness syndrome, NITs），其特征是血清总三碘甲状腺原氨酸（TT3）、游离三碘甲状腺原氨酸（FT3）水平降低，反三碘甲状腺原氨酸（rT3）水平升高，而促甲状腺素（TSH）水平正常，严重病例可以出现总甲状腺素（TT4）和游离甲状腺素（FT4）减低，TSH仍然正常，称为低T3-T4综合征。研究发现，LT3S是恶性肿瘤、严重营养不良、慢性心力衰竭、肾衰竭、败血症、肝病、糖尿病等疾病的独立预后因子[5]–[11]，在血液系统肿瘤的研究中发现，LT3S在慢性淋巴细胞白血病、弥漫大B细胞淋巴瘤中具有预后价值[12]–[13]。然而，关于LT3S在MM中的意义目前报道较少[14]。本研究拟分析LT3S与MM患者临床特征及预后的相关性，以探讨LT3S在MM患者中的临床意义。

## 病例与方法

1. 病例：回顾性分析2009年7月至2020年12月江苏省人民医院血液科诊治的具有详细甲状腺功能资料及完整随访资料的211例初诊MM患者。MM的诊断标准参照《中国多发性骨髓瘤诊治指南（2022年修订）》[15]。排除既往有甲状腺基础疾病、严重肝肾功能障碍、心脑血管疾病、败血症、其他恶性肿瘤、正在服用甲状腺素片及其他含碘药物的患者及资料不全者。本研究经江苏省人民医院医学研究伦理委员会批准（2020-SR-589），并获得患者知情同意。

2. 甲状腺激素检测方法：患者首次入院时进行的血清甲状腺激素水平测试包括FT3、FT4、TSH、TT3、TT4、抗甲状腺过氧化物酶、抗甲状腺球蛋白。以上甲状腺功能指标通过化学发光标记免疫分析法进行测定。将血清FT3水平降低、血清FT4和TSH水平降低或正常定义为LT3S。

3. 随访：通过查阅门诊或住院病历及电话进行随访，随访截至2021年10月31日，中位随访时间37个月。无进展生存（PFS）期定义为确诊至疾病进展、复发或死亡的时间，总生存（OS）期定义为确诊至死亡或随访截止的时间。

4. 统计学处理：应用SPSS 25.0和Graphpad Prism 8.0软件进行统计学分析。符合正态分布的计量资料采用均数±标准差表示，不符合正态分布的资料采用中位数和四分位数间距表示，计数资料采用百分比表示。患者临床特征的比较采用Mann-Whitney *U*检验、*χ*^2^检验或Fisher精确概率法；采用Kaplan-Meier法绘制生存曲线，显著性检验采用Log-rank法；多因素分析采用Cox比例风险模型，使用ROC曲线评估LT3S预测准确性。*P*<0.05为差异有统计学意义。

## 结果

1. 一般临床特征：211例患者中男119例、女92例，中位年龄60（33～86）岁，伴LT3S的患者50例（23.7％），不伴LT3S的患者161例（76.3％）。两组患者的年龄、M蛋白类型、M蛋白定量、DS分期、R-ISS分期、乳酸脱氢酶水平和高危细胞遗传学异常比例等差异均无统计学意义（*P*值均>0.05）。与不伴LT3S患者相比，伴LT3S组患者初诊时β_2_-微球蛋白、C-反应蛋白和血肌酐水平明显升高（*P*值均<0.001），血红蛋白、血小板和血清白蛋白水平显著降低（*P*值分别为<0.001、0.018、<0.001），ISS分期Ⅲ期比例更高（*P*<0.001）（表1）。所有患者均采用硼替佐米和（或）免疫调节剂（沙利度胺或来那度胺）为基础的方案进行诱导治疗，两组患者在诱导治疗方案方面基本平衡（*P*＝0.737）。

**表1:** 伴低T3综合征和不伴低T3综合征的初诊多发性骨髓瘤患者的临床特征比较

临床特征	总体（211例）	伴LT3S组（50例）	不伴LT3S组（161例）	统计量	*P*值
男性[例(％)]	119（56.4）	19（38.0）	100（62.1）	9.020	0.003
年龄（岁，*x±s*）	60.24±9.25	61.62±9.84	59.81±9.05	1.170	0.243
M蛋白[例(％)]				0.184	0.980
IgG	100（47.4）	12（24.0）	77（47.8）		
IgA	40（19.0）	9（18.0）	31（19.3）		
轻链型	56（26.5）	14（28.0）	42（26.1）		
其他	15（7.1）	4（8.0）	11（6.8）		
DS分期[例(％)]				2.647	0.266
Ⅰ期	13（6.2）	4（8.0）	9（5.6）		
Ⅱ期	26（12.3）	3（6.0）	23（14.3）		
Ⅲ期	172（81.5）	43（86.0）	129（80.1）		
ISS分期[例(％)]				15.818	<0.001
Ⅰ期	36（17.1）	2（4.0）	34（21.1）		
Ⅱ期	76（36.0）	13（26.0）	62（38.5）		
Ⅲ期	99（46.9）	35（70.0）	64（39.8）		
R-ISS分期[例(％)]				5.415	0.067
Ⅰ期	15（9.0）	1（2.6）	14（10.9）		
Ⅱ期	118（70.7）	25（65.8）	93（72.1）		
Ⅲ期	34（20.3）	12（31.6）	22（17.0）		
FT3（pmol/L，*x±s*）	4.113±1.486	2.259±0.585	4.688±1.177	−19.553	<0.001
FT4（pmol/L，*x±s*）	15.615±2.896	14.230±2.559	16.045±2.866	−4.006	<0.001
TSH（mIU/L，*x±s*）	2.207±1.131	2.347±1.003	2.163±1.167	1.003	0.317
M蛋白 [g/L，*M*（*IQR*）]	11.20（0～35.53）	8.16（0～32.38）	15.70（0～38.60）	−0.664	0.508
β_2_-微球蛋白 [mg/L，*M*（*IQR*）]	4.77（3.12～10.02）	10.40（5.02～23.45）	4.23（2.96～7.48）	−5.069	<0.001
血清白蛋白（g/L，*x±s*）	33.56±7.56	30.31±6.84	34.57±7.51	−3.574	<0.001
乳酸脱氢酶 [U/L，*M*（*IQR*）]	176.00（147.00～229.00）	177.50（138.00～270.75）	176.00（149.00～226.50）	−0.719	0.472
PLT [10^9^/L，*M*（*IQR*）]	158.00（123.50～217.50）	140.50（90.50～181.25）	168.50（129.50～233.00）	−2.368	0.018
HGB[g/L，*M*（*IQR*）]	88.00（71.00～111.25）	71.00（60.00～84.00）	97.50（77.00～116.75）	−5.813	<0.001
C-反应蛋白 [mg/L，*M*（*IQR*）]	3.28（3.13～10.63）	7.17（3.22～22.73）	3.25（3.04～6.26）	−3.503	<0.001
血肌酐[例(%)]				20.158	<0.001
<177 µmol/L	163（77.3）	27（54.0）	136（84.5）		
≥177 µmol/L	48（22.7）	23（46.0）	25（15.5）		
高危细胞遗传学异常[例(％)]	50（56.8）	12（63.2）	38（55.1）	0.397	0.529
1q扩增	44（50.0）	10（52.6）	34（49.3）	0.067	0.796
del（17p）	8（9.1）	1（5.3）	7（10.1）	0.430	0.512
t（4;14）	8（9.1）	1（5.3）	7（10.1）	0.430	0.512
诱导治疗方案[例(％)]				0.113	0.737
硼替佐米为基础的方案	164（77.7）	38（76.0）	126（78.3）		
免疫调节剂为基础的方案	47（22.3）	12（24.0）	35（21.7）		

注 FT3：游离三碘甲状腺原氨酸；FT4：游离甲状腺素；TSH：促甲状腺素

2. 生存和预后分析：伴LT3S组与不伴LT3S组患者的中位PFS期分别为16个月和30个月，中位OS期分别为57个月和75个月，伴LT3S组的PFS期及OS期均较不伴LT3S组缩短，差异均有统计学意义（*P*值分别为0.003和0.004）（图1）。对211例患者的预后进行单因素分析，纳入因素包括性别、年龄、β_2_-微球蛋白、血清白蛋白、血红蛋白、血肌酐、R-ISS分期和LT3S，由于R-ISS分期包括ISS分期、乳酸脱氢酶和高危细胞遗传学异常，因此只有R-ISS分期被纳入分析。将*P*<0.05的因素纳入Cox比例风险模型进行多因素分析。结果显示，影响患者PFS的独立不良预后因素包括R-ISS分期Ⅲ期（*HR*＝1.805，95％ *CI* 1.044～3.119，*P*＝0.034）和LT3S（*HR*＝2.114，95％ *CI* 1.271～3.516，*P*＝0.004），影响患者OS的独立不良预后因素为LT3S（*HR*＝2.231，95％ *CI* 1.088～4.577，*P*＝0.029）（表2）。

**图1:**
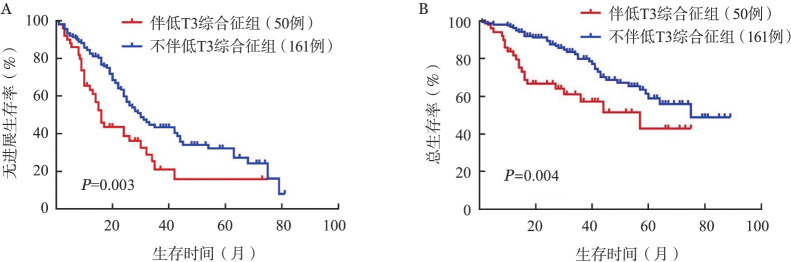
伴低T3综合征和不伴低T3综合征的多发性骨髓瘤患者的无进展生存（A）、总生存（B）曲线

**表2:** 211例初诊多发性骨髓瘤患者预后的危险因素分析

因素	无进展生存				总生存			
	单因素分析		多因素分析		单因素分析		多因素分析	
	*HR*（95% *CI*）	*P*值	*HR*（95% *CI*）	*P*值	*HR*（95% *CI*）	*P*值	*HR*（95% *CI*）	*P*值
男性	0.943（0.656~1.357）	0.753			0.925（0.556~1.540）	0.765		
年龄≥65岁	1.005（0.985~1.026）	0.605			1.040（1.010~1.071）	0.008	1.033（0.989~1.078）	0.141
β_2_-微球蛋白≥3.5 mg/L	2.015（1.285~3.160）	0.002	1.795（0.983~3.279）	0.057	2.290（1.160~4.519）	0.017	1.512（0.555~4.121）	0.419
血清白蛋白<35 g/L	0.928（0.643~1.339）	0.689			0.894（0.536~1.491）	0.669		
HGB<85 g/L	1.244（0.866~1.788）	0.238			1.699（1.019~2.833）	0.042	0.930（0.463~1.866）	0.838
血肌酐≥177 µmol/L	1.577（1.049~2.371）	0.029	0.930（0.513~1.685）	0.810	1.527（0.868~2.687）	0.142		
R-ISS分期Ⅲ期	2.273（1.359~3.804）	0.002	1.805（1.044~3.119）	0.034	2.363（1.163~4.803）	0.017	2.071（0.993~4.320）	0.052
低T3综合征	1.792（1.204~2.668）	0.004	2.114（1.271~3.516）	0.004	2.164（1.271~3.685）	0.004	2.231（1.088~4.577）	0.029

采用ROC曲线对患者的FT3进行分析，结果显示FT3预测PFS的曲线下面积为0.732（95％ *CI* 0.664～0.801，*P*<0.001），预测OS的曲线下面积为0.670（95％ *CI* 0.587～0.752，*P*<0.001），预测PFS和OS的最佳临界值分别为4.28 pmol/L和4.26 pmol/L（图2）。

**图2:**
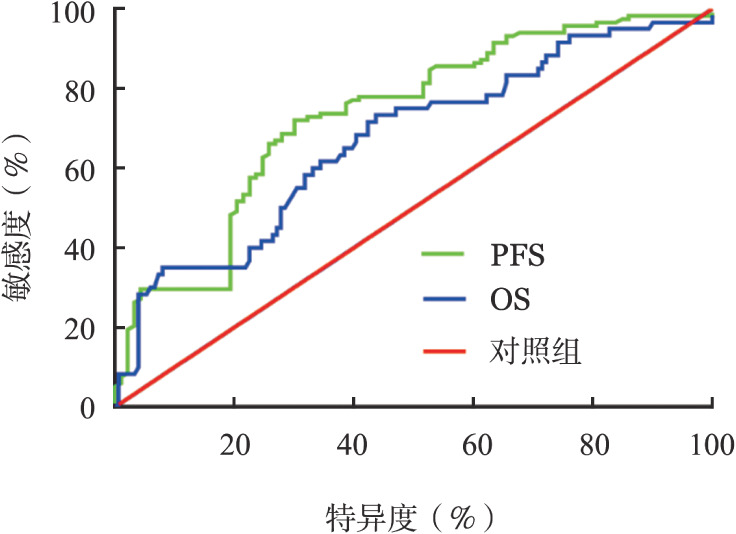
游离三碘甲状腺原氨酸预测多发性骨髓瘤患者无进展生存（PFS）期、总生存（OS）期的ROC曲线

3. FT3、FT4与伴LT3S的MM患者生存结局的相关性：50例患者在首次入院时被诊断为LT3S，其中11例患者FT3和FT4同时降低，其余39例患者仅FT3水平降低。我们对仅FT3降低和伴FT3和FT4同时降低患者的PFS和OS进行比较，发现FT3和FT4同时降低的患者具有更差的OS（*P*＝0.001），但是PFS差异无统计学意义（*P*＝0.318）（图3）。表明FT3和FT4同时降低可以作为伴LT3S的MM患者死亡结局的预测因子。

**图3:**
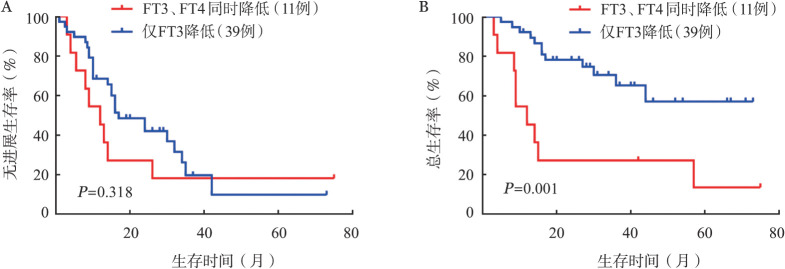
仅FT3降低和FT3、FT4同时降低的多发性骨髓瘤患者的无进展生存（A）、总生存（B）曲线 注 FT3：游离三碘甲状腺原氨酸；FT4：游离甲状腺素

## 讨论

甲状腺激素是人体重要的内分泌激素之一，具有促进生长发育、提高新陈代谢等作用，与机体脏器功能密切相关。LT3S是由于严重的全身性疾病导致血液循环中甲状腺激素水平减低，是机体的一种保护性反应，而非甲状腺原发病病变。在过去二十年中，多项研究报道LT3S在危重患者中非常常见，在严重创伤、终末期肾病、急性脑卒中、呼吸衰竭、社区获得性肺炎和急性失代偿性心力衰竭中的发生率分别为90.0％、78.6％、56.6％、53.1％、31.8％和28.3％[11],[16]–[20]。在弥漫大B细胞淋巴瘤、慢性淋巴细胞白血病中LT3S的发生率分别为12.8％和14.3％[12]–[13]，Pan等[14]报道了201例MM患者中LT3S的发生情况，结果显示80例（39.8％）发生LT3S。本研究中，LT3S的发生率为23.70％，略低于Pan等的报道，但高于弥漫大B细胞淋巴瘤和慢性淋巴细胞白血病，这可能与MM患者发病年龄高、基础疾病较多有一定关联。

LT3S的发生与甲状腺激素受体、外周甲状腺激素摄取、皮质类固醇和细胞因子的变化等因素有关，白细胞介素1（IL-1）、肿瘤坏死因子（TNF-α）及白细胞介素6（IL-6）等炎性因子大量分泌，使5′脱碘酶活性受到抑制，导致T3生成减少，向rT3代谢增多[21]–[22]，且大量失控的炎症介质可进一步消耗T3。此外，肝脏的Ⅰ型脱碘酶活性受到抑制使得T4外环脱碘向T3转换减少，而活性增强的Ⅲ型脱碘酶则可促使T4向rT3转换，二者共同作用促进了LT3S的发生[23]。在化学和细菌性炎症期间，Ⅲ型脱碘酶在浸润性粒细胞的杀菌作用中也起着重要作用[24]，在危重病的急性期，LT3S通过负反馈调节来保存能量和抵消过度的分解代谢[25]，当危重疾病发展到慢性阶段，高分解代谢失调反应降低了线粒体活性，导致肌肉功能障碍，致使低T3状态变得对患者尤为不利[26]。MM是慢性消耗性疾病，炎症和蛋白质营养不良等多种因素可能导致LT3S的发展，MM中血清白蛋白水平降低时，LT3S也可能被诱发作为减少能量消耗的保护机制。目前还不清楚LT3S是否还存在其他途径在MM中发挥作用，有待于进一步研究进行深入探索。

在本研究中，我们发现伴LT3S患者的β_2_微球蛋白、血肌酐和C反应蛋白水平较高，而血红蛋白、血小板和血清白蛋白水平较低，ISS分期Ⅲ期比例更高。既往文献报道LT3S可能是急性脑血管疾病[19]、慢性心力衰竭[20]、终末期肾病[25]中不良结局的独立预后因素，在慢性淋巴细胞白血病的研究中，LT3S是患者诊断到治疗时间、肿瘤特异性生存时间的独立预后因素[12]，在弥漫大B细胞淋巴瘤和多发性骨髓瘤研究中，LT3S是患者PFS、OS的独立预后因素[13]–[14]，我们的研究结果与此一致，T3水平低的MM患者比T3水平正常的患者预后更差，LT3S也是MM患者PFS和OS的独立预后因素，与FT4和TSH相比，FT3对不良结局具有更好的预测能力。既往文献报道LT3S合并低T4状态的患者病情更重，预后更差，死亡率明显增加[27]，在本研究中我们同样发现，FT3和FT4同时降低的患者具有更差的OS，因此我们推测同时具有低FT3和低FT4水平的患者生存更差，低FT4水平可以作为伴LT3S的MM患者死亡结局的预测因子。LT3S作为一种预后标志，具有检测便捷、价格低、不受饮食影响等显著优势。

总之，LT3S与MM患者的不良结局密切相关，并且是独立的不良预后因素；FT3和FT4同时降低的患者生存期最差，FT4可以进一步识别伴LT3S的MM患者中的高危病例。由于本研究属于单中心回顾性研究，存在研究人群单一、样本量较少等问题，且我们仅在初诊时对患者进行甲状腺功能的评估，未在整个随访期间进行甲状腺功能监测，故存在一定局限性，未来我们还需要扩大样本和延长随访期以及动态评估甲状腺功能，以进一步明确LT3S作为不良预后预测因子的作用。
